# Effects of truck-mounted, ultra low volume mosquito adulticides on honey bees (*Apis mellifera*) in a suburban field setting

**DOI:** 10.1371/journal.pone.0193535

**Published:** 2018-03-01

**Authors:** Vivek Pokhrel, Nicholas A. DeLisi, Robert G. Danka, Todd W. Walker, James A. Ottea, Kristen B. Healy

**Affiliations:** 1 Department of Entomology, Louisiana State University Agriculture Center, Baton Rouge, LA, United States of America; 2 St. Tammany Parish Mosquito Abatement District, Slidell, LA, United States of America; 3 USDA-ARS Honey Bee Breeding, Genetics, and Physiology Laboratory, Baton Rouge, LA, United States of America; 4 East Baton Rouge Parish Mosquito Abatement and Rodent Control, Baton Rouge, LA, United States of America; Institut Sophia Agrobiotech, FRANCE

## Abstract

Few studies have examined the impact of mosquito adulticides on honey bees under conditions that reflect actual field exposure. Whereas several studies have evaluated the toxicity of mosquito control products on honey bees, most have been laboratory based and have focused solely on acute mortality as a measure of impact. The goal of this study was to determine effects of routine applications of truck-based ultra-low volume (ULV) mosquito adulticides (i.e., Scourge, Duet, and Deltagard) on honey bees in a suburban setting. The mosquito adulticides used in this study were pyrethroids with active ingredients resmethrin (Scourge), prallethrin and sumithrin (Duet), and deltamethrin (Deltagard), in which resmethrin, prallethrin, and sumithrin were synergized with piperonyl butoxide. We measured and compared mortality and detoxification enzyme activities (esterase and glutathione *S*-transferase) from sentinel beehives within and outside of mosquito control areas. Concurrently, colony health (i.e., number of adult bees, brood quantity and brood quality) was compared throughout the study period. No significant differences were observed in honey bee mortality, colony health or detoxification enzyme activities between treated (five sprayed areas each received one to three insecticide treatment) and control sites (four unsprayed areas that did not receive insecticide treatment) over the seven week study period. However, our laboratory study showed that exposure to resmethrin, the active ingredient in Scourge, caused significant inhibition of esterase activity compared with the control group. Our findings suggest that proper application of truck based insecticides for mosquito control results in little or no exposure and therefore minimal effects on domestic honey bees.

## Introduction

Recent increased loss of managed honey bees, *Apis mellifera* L [1], has raised concern regarding potential causes. Whereas it is likely that there are multiple factors associated with colony losses, pesticides have been shown to affect several parameters of honey bee health [2–5]. Although effects of agricultural pesticides on honey bees are fairly well-studied, there have been few reports evaluating non-target effects of public health pesticides on honey bees [6–8].

Among several approaches to managing mosquitoes, chemical control is the most efficient measure adopted by mosquito control professionals when there is high risk of mosquito nuisance and arbovirus prevalence. The use of Ultra Low Volume (ULV) mosquito adulticides is common in urban areas because judiciously applied chemicals are highly effective in controlling mosquitoes while having little impact on non-target organisms [9]. The improper application of mosquito adulticides can result in acute honey bee mortality, but most studies have shown minimal impacts when applications are made at the label rate during night time when honey bees are within the hive [10–12]. Most research on the effect of mosquito adulticides on honey bees has focused on acute mortality following exposure of caged bees to insecticides [13,14]. Although acute mortality is an important indicator of pesticide exposure, other, chronic and sublethal effects on colony health should also be evaluated [15–17]. In addition, most studies have not utilized field realistic scenarios with respect to foraging patterns of bees or actual field exposure to insecticides.

Activities of detoxification enzymes have been evaluated as biomarkers for insecticide exposure in honey bees and other insects [18–20]. Esterases and glutathione *S-*transferases (GSTs) are enzymes used by insects to detoxify insecticides [21–23]. Esterases detoxify many organophosphate and pyrethroid insecticides (some of which are used as mosquito adulticides) by hydrolyzing the ester moieties and making products that are more hydrophilic and less toxic [24]. Similarly, GSTs detoxify xenobiotics (including some insecticides) by accelerating the reaction between reduced glutathione and electrophilic centers, creating products with decreased lipophilicity and toxicity [25,26]. GSTs also play a role in antioxidant defense and ameliorate effects of oxidative stress from exposure to insecticides [27]. Exposure to xenobiotics including insecticides alter the detoxifying enzyme activities both by induction and inhibition [28]. We hypothesize that insecticides exposure would decrease the esterase and GSTs activities in honey bees in our study Activities of these enzymes have been measured in response to exposure of honey bees to insecticides [18,29], but to date, have not been evaluated as biomarkers for exposure to mosquito adulticides.

The main goal of this study was to examine the effects of truck based ULV mosquito adulticides on acute mortality, colony populations, and detoxification enzymes of honey bees in a field setting. The utility of measuring esterase and GST activities as indicators of exposure to pyrethroid insecticide was validated in the laboratory, and examined in the field setting. In addition, acute mortality and colony health following insecticide exposure were measured weekly at field sites receiving routine ULV sprays for seven weeks and compared with unsprayed sites. Results from this study can help improve best management practices for mosquito control to reduce impact to non-target organisms, such as honey bees.

## Materials and methods

### Ethics statement

No specific permits were required for the field studies, and all work in the field was done with homeowner consent. These studies did not involve endangered or protected species.

### Chemicals

Sodium phosphate monobasic monohydrate (≥98%), sodium phosphate dibasic heptahydrate (98%), Brilliant Blue G-250 (ultra pure), dimethyl sulfoxide (≥99%), Fast Blue B salt (approx. 95%), L-glutathione, reduced (≥98%), 1-naphthyl acetate (*α* NA) (≥98%), 1 chloro 2,-4 dinitrobenzene (CDNB, 98%), and resmethrin (analytical standard, 99.4%) were purchased from Sigma Aldrich (St. Louis, MO). Phosphoric acid (85%), hydrochloric acid (99.7%), sodium dodecyl sulfate (SDS) (99%), and sodium hydroxide (ACS grade) were purchased from Fisher Scientific (Kansas City, MO). Bovine serum albumin (biotechnology grade) and acetone (ACS grade) were purchased from Amresco (Solon, OH). Ethyl alcohol (absolute; ACS/USP grade) was purchased from Pharmco-Aaper (Brookfield, CT).

### Field experimental sites

**Fig 1:**
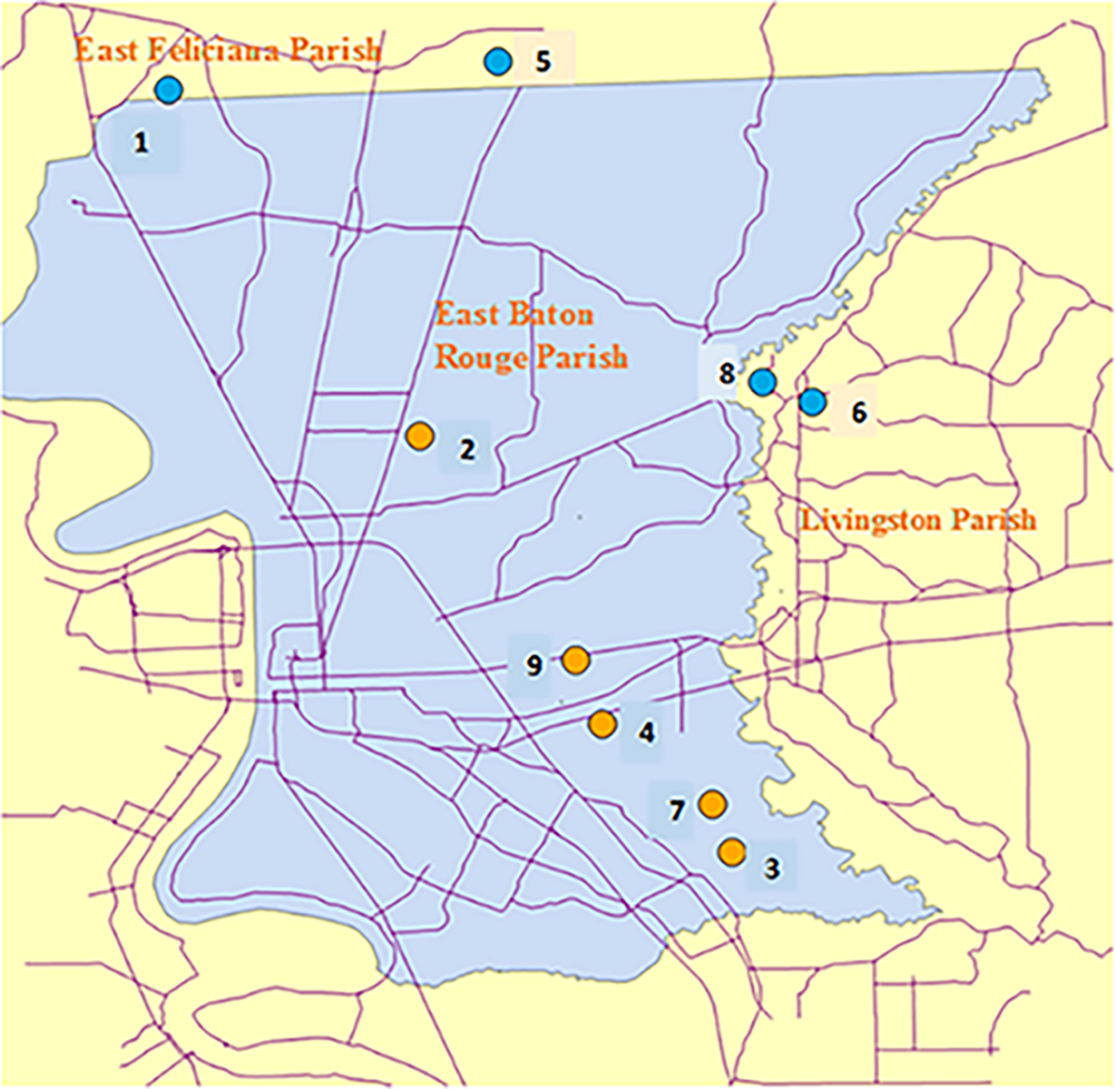
Experimental sites used for study. Blue circles represent control sites and orange circles represent treatment sites (ArcMap 10.2; ESR1).

The experimental sites were selected with the coordination of the Louisiana Beekeepers Association, Capitol Area Beekeepers Association and the East Baton Rouge Mosquito Abatement and Rodent Control District (EBRMARC), (Baton Rouge, LA; Fig 1). Local beekeepers volunteered the use of their colonies for this study. Five sites that were routinely sprayed with mosquito adulticides were designated treated sites, and four sites that were never sprayed were designated control sites. Three colonies were used at each experimental site, for a total of 12 control and 15 treatment colonies. However, two colonies at one of the control sites (site 6) were excluded from data analysis because both adults and brood were completely absent (for unknown reason) at the end of the study. The study was conducted over a seven-week period from 7 August to 25 September 2015. This time period was selected due to the high spraying frequency for mosquito control during this part of the year.

### Insecticides sprayed in the field

At each of the five treatment sites, ULV applications of pyrethroid insecticides were made just after sunset (between 7:00 pm to 10:00 pm) in response to local mosquito control needs (Table 1). These treatments were administered by EBRMARC personnel using a truck mounted, ULV sprayer. At each location, insecticide droplets were collected using two Teflon coated slides (26mm × 76mm) mounted on spinners (Leading Edge, Fletcher, NC) placed at 15.2 m and 30.5 m from the roads where sprays originated. Spinners were mounted 0.3 m above the ground. One or two spinners were also set near the experimental hives, which were at the distances of 56 m to 253 m (average 117 m; control sites) and 44 m to 92 m (average 62 m; treated sites) from the point of spray. Sites at which all hives were equidistant from the road had one spinner near the hives, whereas those not equidistant had two spinners. Spinners were fixed at least 2 hours before an insecticide application. All slides were collected from sites in the early morning after the application. In preliminary tests, we found no difference in volume of droplets (measured as Volume Mean Diameter; VMD) between the slides collected at 2 hours and 12 hours post spray. Control spinners were set using similar methods, at locations not receiving mosquito control.

**Table 1:** Spray history at treated sites.

Spray date (2015)	Site	Chemicals used	Application Rate
Aug. 20	3,4 and 7	Duet	a) Deltagard = 0.00045 lb/acre of deltamethrin b) Scourge = 0.002 lb/acre of resmethrin and 0.0059 lb/acre piperonyl butoxide (PBO) c) Duet = 0.00036 lb/acre prallethrin, 0.0018 lb/acre sumithrin, and 0.0018 lb/acre piperonyl butoxide (PBO)
Aug. 31	3 and 7	Scourge and Deltagard	
Sep. 1	2	Scourge	
Sep.11	2	Scourge	
Sep. 14	8	Scourge	
Sep. 15	3,4 and 7	Duet, Scourge, and Deltagard	
Sep. 21	2	Scourge	

Insecticide droplets were analyzed at EBRMARC using Drop Vision software (Version 2.4, Leading Edge). The number and diameter of droplets were calculated by making thirty measurements per slide, which typically resulted in over 200 droplets per slide at our treated sites. Droplets were also measured from slides at sites that were not sprayed (control). Typically control slides contained less than 30 droplets per slide. Volume Mean Diameter (VMD) was measured to one micron using the Drop Vision software. In order to account for other environmental droplets such as morning dew on the slides, we adjusted all droplet data to a standard area (200 sq. cm). Frequency of treatment droplets was then calculated by subtracting the number of control droplets for each droplet diameter. The frequency was then multiplied by the diameter size to determine a volume. This was then divided by the total volume on the corrected treatment slide. The VMD was then calculated as the droplet diameter in which 50% of the cumulative volume was reached. These measurements are generally made in an attempt to verify that insecticide is reaching the target insects and their habitat [9]. This method is often used in mosquito control program during ULV spraying [30]. Our assumption is that dramatic difference measured in droplets between control and treated sites was due to insecticide deposition. However, insecticides residues were not analyzed.

### Acute bee mortality

Estimates of adult bee mortality were based on collection from a dead bee trap, which was designed for this study (Figure in S1 Appendix). This trap, designated the MHH trap, was modified from designs described by Hendrkisma and Hatrel [31]. Dead bee traps were fixed onto all experimental hives a week prior to the study in order to acclimatize the bees, then dead bees from each colony were collected and counted weekly throughout the seven weeks of the experimental period. The MHH traps were emptied and cleaned after each collection period.

### Colony health

Colony health was assessed in treated and control colonies at the beginning and end of the study period. Parameters used to measure colony health were number of adult bees, brood quantity, and brood quality. Brood quantity reflects the overall health and productivity of a colony through the measure of colony strength (population), whereas brood quality represents the status of queen health (inbreeding between queen and her mates, the susceptibility of colony to brood diseases) [32]. Estimates of these parameters were made by a single observer (VP) to minimize the error from individual bias throughout the study. Numbers of adult bees were calculated using methods described by Burgett and Burikam [33], in which number of adults on both sides of individual combs were estimated. Similarly, brood quantity was measured by determining the surface area covered by capped brood on both sides of each frame in the hives [32]. Percentage area covered by capped brood was converted into the area of brood in square centimeters, to correct for the difference in frame sizes. For measurement of brood quality, a rhombus-shaped plastic grid (measured as 10 by 10 honey bee cells) was used. The grid was placed on selected frames with large patches of capped brood, and the number of empty cells was recorded and expressed as an average of three measurements per hive.

### Enzyme activities in adult honey bees

In order to compare enzyme activity between treated and control sites, an average of ten live forager bees was collected randomly from each experimental hive weekly from August 7 to September 26, 2015. In addition, ten forager bees were collected randomly from the treated sites immediately before (pre-spray) and within 12 hours after (post-spray) a mosquito control application. All bees were transferred within hours to the laboratory in an ice-filled cooler, and kept in a -80°C freezer until enzyme assays.

To validate use of esterase and GST activities as biomarkers for insecticide exposure, adult bees were treated with a sublethal dose of resmethrin in the laboratory. Ten foragers of mixed age were collected from a colony that was maintained at Louisiana State University, and were placed into 475-ml wax-paper cups that were covered with nylon tulle that was secured with a rubber band. Individual bees from three replicate cups were CO2 anesthetized and treated topically on the thoracic dorsum with 1 μl of resmethrin solution (in acetone; 0.013 μg resmethrin/bee) using a 50-μl syringe with a mechanical repeating dispenser (Hamilton Company, Reno, NV). The dose used for this study was determined from preliminary bioassays in which mortality was < 2%. Control bees were handled the same as the treated bees except they were treated with 1 μl of acetone only. All bees were kept in an incubator (33°C with 75% humidity) for 12 hrs and provided with 50% sucrose solution. After 12 hrs. bees were removed and placed in a -85°C freezer for 24 hours. After 24 hours, bees were thawed and used immediately for biochemical studies.

### Measurement of detoxifying enzyme activities

Abdomens were removed from frozen bees and homogenized in 0.1 M sodium phosphate buffer (pH 7.4; 1 bee/500 μl) using 10 strokes of an all glass homogenizer. Homogenates were centrifuged at 4°C for 10 min at 14,600 rpm. Resulting supernatants were held in ice and diluted two-fold with buffer to adjust protein for enzyme assays. Preliminary assays were used to optimize pH and protein concentration (data not shown).

Activities of esterase towards alpha naphthyl acetate (αNA) were measured using the method of Gomori [34] as modified by van Asperen [35] and Grant [36] in polystyrene 96-well flat bottom microplates (Costar, Cambridge, MA). All microplates were prewashed with 2.5% Tween 20 (v/v in water). A stock solution of *α*NA (30 mM) in acetone was diluted in buffer to a concentration of 0.3 mM. Reactions, containing 20 μl of either enzyme homogenate (0.02 insect equivalent; 0.0044 mg protein) or buffer, were started by adding 200 μl of αNA (0.27 mM, final concentration). After 10 mins at 27°C, reactions were terminated by addition of 50 μl of Fast Blue B dye (0.15 gm Fast Blue B salt + 14 ml distilled water + 30 ml 5% SDS solution; 2.18 mM final concentration). Reactions with buffer only were used as control. Optical density of reactions, measured at 570 nm using a Thermomax microplate reader (Molecular Devices, Palo Alto, CA), was converted to μmol/min using an experimentally derived extinction coefficient of 0.0235 μM^-1^ 250 μl for alpha naphthol.

Activities of GST towards 1-chloro-2,4-dinitrobenzene (CDNB) were measured following the method of Booth *et al*. [25] and Jakoby [37], as modified by Grant *et al*. [36]. A stock solution of CDNB (50 mM in DMSO) was diluted in buffer to a concentration of 0.66 mM. Glutathione (65 mM) was prepared in 0.1 M sodium phosphate buffer, pH 7.4. A typical reaction mixture consisted of 20 μl of enzyme homogenates (0.02 insect equivalent; 0.0044 mg protein) or buffer (control), 30 μl of glutathione (7.8 mM final concentration) and 200 *μl* of CDNB (0.53 mM final concentration). Rate of change in optical density was measured for 10 mins at 340 nm using a Thermomax microplate reader (Molecular Devices, Palo Alto, CA) and first order reaction rates were converted to pmol/min using the experimentally derived extinction coefficient of 8.39 m*M^-1^* 250 μl for conjugated CDNB [36]. Protein concentrations were measured using the method of Bradford [38], using bovine serum albumin as the standard.

### Statistics

The number of dead bees collected was converted into percentage mortality of the estimated total numbers of bees in the colony followed by arcsine transformation of percentage data. The Proc Mixed one way Analysis of Variance (SAS Institute, Cary, NC, 2013) was used to compare the percentage of dead bees between treated and control colonies. Means were compared at α < 0.05 by Tukey’s Honest Significant Difference test.

Number of adult bees, brood quantity and brood quality were converted into percentage changes with the general formula (% change = (initial reading–final reading)/ initial reading × 100) and subsequently percentage data were arcsine transformed. Proc t-test (SAS institute, Cary, NC, 2013) was used to compare the percentage change in number of adult bees and brood quality between control and treatment colonies during the experimental periods. Due to non-normal distribution of brood quantity data, Mann Whitney’s test was used to compare the percentage change in brood quantity between control and treatment colonies.

Proc Glimmix Repeated Anova (SAS institute, Cary, NC, 2013) was used to analyze differences in enzyme activities between two treatments. Tukey-Kramer (P < 0.05) method was used to compare enzyme activities between before and after mosquito control applications.

## Results

### Effects of insecticide sprays on honey bee mortality and colony health

**Fig 2:**
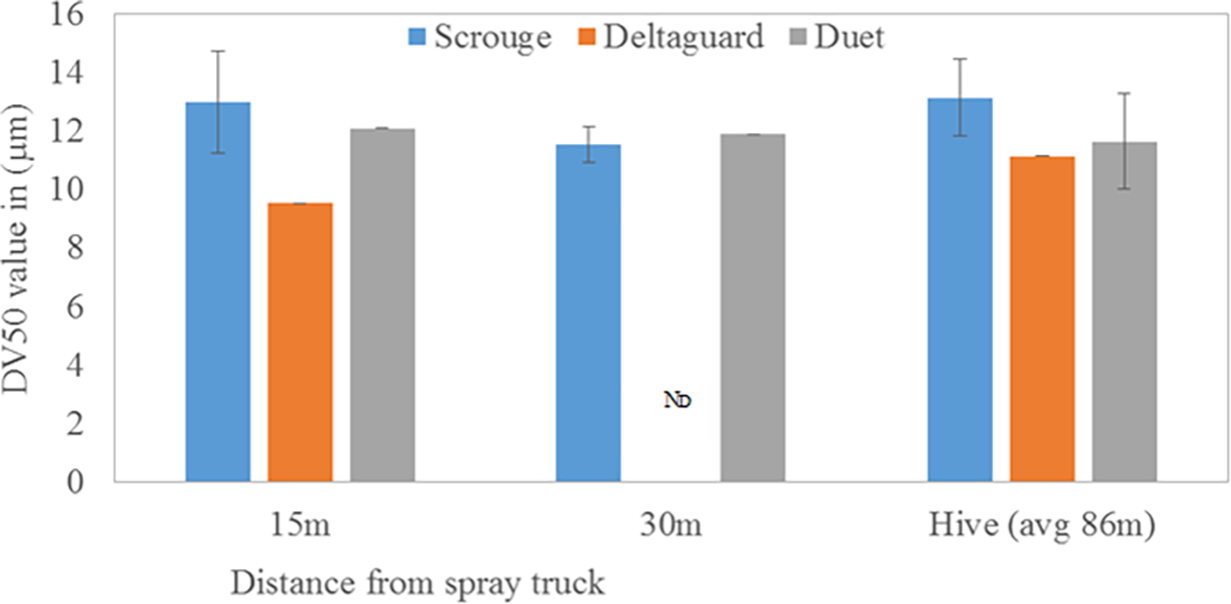
Insecticide droplet size measured from treated sites at different distances from point of release of insecticides from spray truck and at experimental hives. Bars represent mean droplet size, expressed as diameter in mean volume (DV_50_) based on 3 determinations replicated 3 times. ND = not determined.

No significant effect on acute honey bee mortality was observed from the routine spray of insecticides by mosquito control program despite apparent exposure of experimental hives to the insecticides sprayed. Based upon deposition of insecticide droplets, exposure at the experimental hives was similar between 50 m and 100 m from the road (Fig 2), with average DV50 values for Scourge^TM^, Deltaguard^TM^, and Duet^TM^ of 12.59, 10.33, and 11.81 microns, respectively. The droplet sizes were in the optimum range used in mosquito control [30]. Bee mortality, averaged from weekly collections from dead bee traps, was low (≤0.33%) for the four untreated (0.33% ± 0.13) and five treated (0.22% ± 0.11) sites, and was not significantly different between these treatment groups (F_1, 6.8_ = 0.51; p = 0.498; Table 2). Although bee mortality varied among the nine experimental sites (ranging from 0.06 to 0.73%), differences were not statistically significant between any two of the individual sites. Similarly, mortality did not differ significantly from week to week (F_6, 133_ = 1.71; P = 0.124) among bees from control and treated colonies (data not shown). Further, numbers of adult bees declined from the beginning to the end of the seven week study period at both control (7.36%) and treated (4.18%) sites, but these decreases were not statistically significant (T_20_ = -0.14; P = 0.886; Table 2). Finally, there was no significant difference (F_9, 9_ = 1.66; P = 0.998) in the percentage change in brood quality between treated and control colonies (Table 2). However, there was a significant increase (U_75, 135_ = 20; P = 0.034) in brood quantity in both treated and control colonies during the seven-week test (Table 2).

**Table 2:** Comparison of weekly bee mortality and colony health among insecticide-treated and–untreated sites.

Parameters	Mean ± SEM		Statistical Test	P value
	Control	Treated		
Weekly Bee Mortality	0.3 ± 0.1a	0.2 ± 0.1a	ANOVA (F_1,6.8_ = 0.51)	0.498
% Change in Bee Number	-7.4 ± 11a	-4.2 ± 17a	T test (T_20_ = -0.14	0.886
% Change in Brood Quality	26 ± 22a	20 ± 22a	T test (T_16.9_ = 0.0025)	0.998
% Change in Brood Quantity	75 ± 10a	135 ± 7.3b	Mann Whitney’s (U_17,135_ = 20)	0.034

Weekly bee mortality (Mean ± SEM) was measured throughout the seven week study. Colony health (number of bees, brood quantity, and brood quality) is expressed as percentage change (Mean ± SEM) based on measurements at the beginning and at the end of the seven week study period. Significant differences between treated and untreated colonies are indicated by different letters.

### Effect of insecticide exposure on enzyme activities

**Fig 3:**
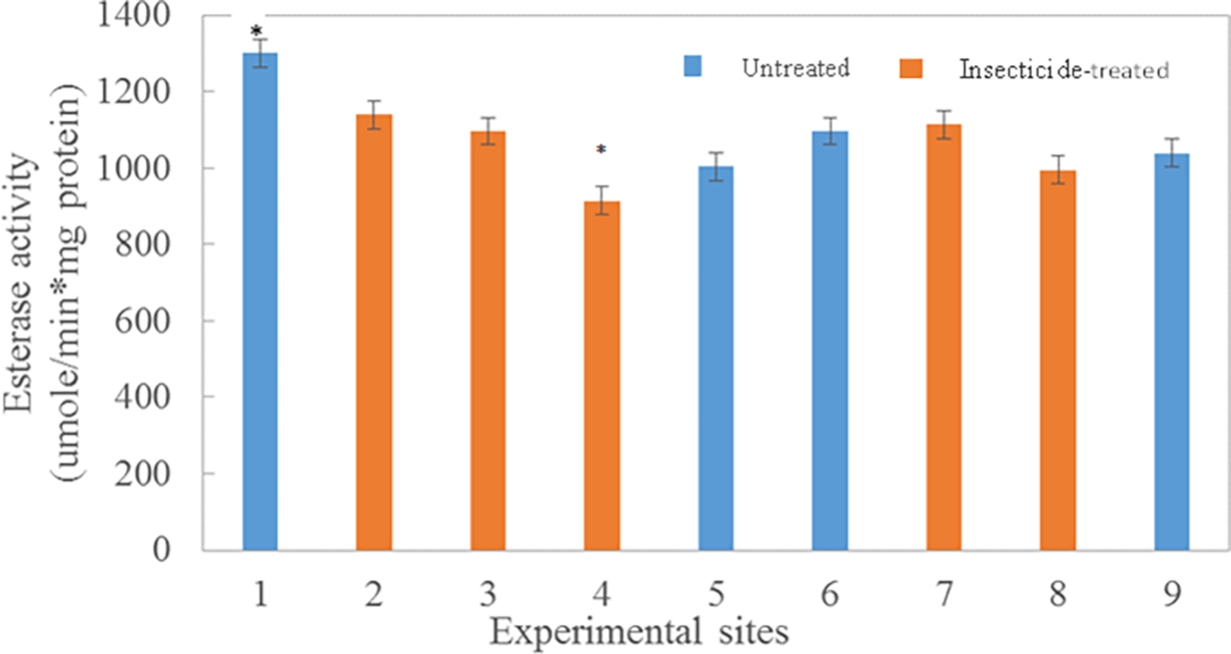
Esterase activity from bees collected during a seven week period from insecticide-treated (orange bars) or–untreated (blue bars) sites. Bars represent mean activities (μmole/min*mg protein; ± SEM) based on triplicate assays made from 10 bees collected weekly at 3 colonies from each treated or untreated site. Asterisks signify mean values that are significantly different (P<0.05).

**Fig 4:**
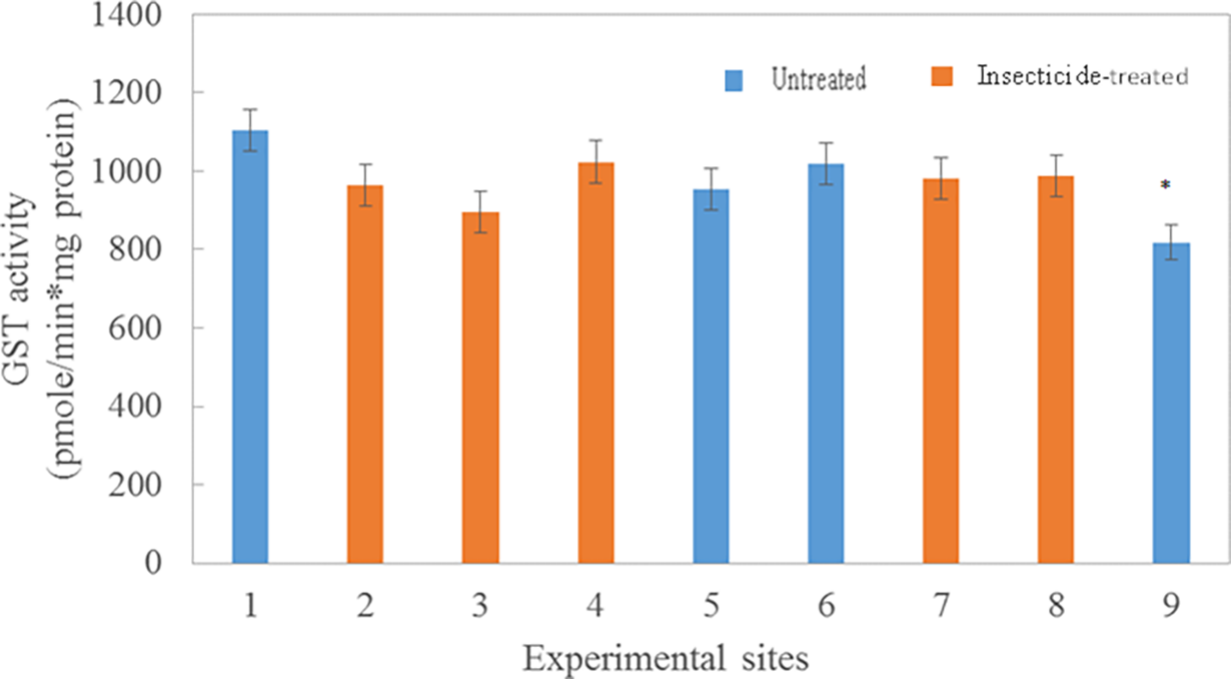
GST activity from bees collected during seven week period from insecticide-treated (orange bars) or–untreated (blue bars) sites. Bars represent mean activities (pmole/min*mg protein; ± SEM) based on triplicate assays made from 10 bees collected weekly at 3 colonies from each treated or untreated site. Asterisks signify mean values that are significantly different (P<0.05).

**Fig 5:**
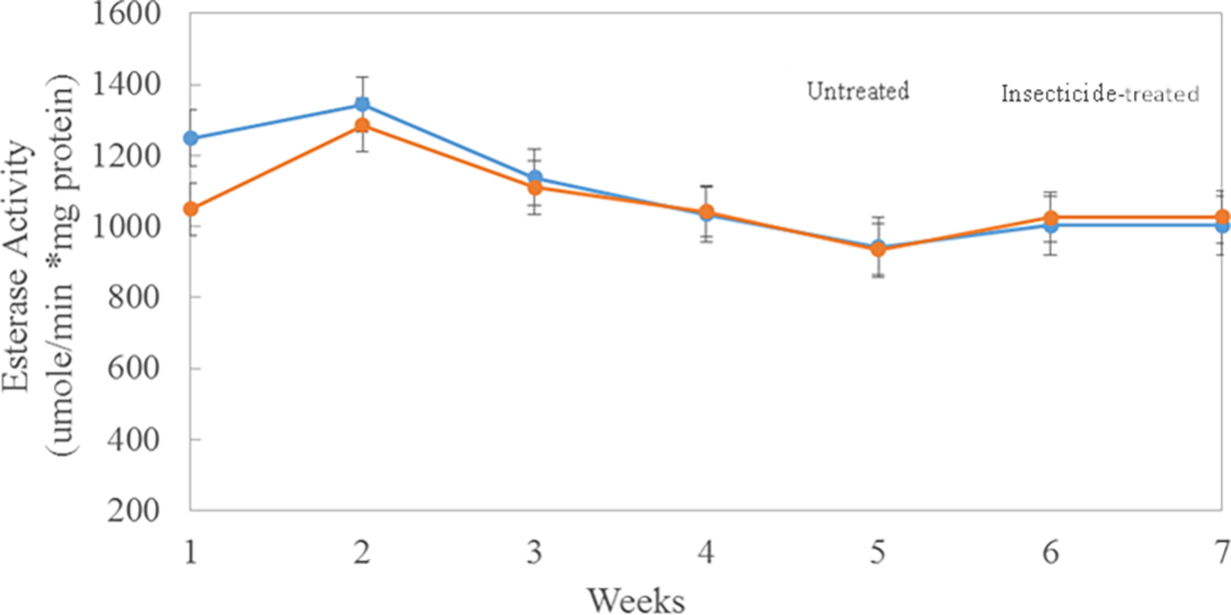
Esterase activity from bees collected weekly from five insecticide-treated (orange line) or–untreated (blue line) sites. Points represent mean activities (μmole/min*mg protein; ± SEM) from triplicate assays of 10 bees collected from 3 colonies from each treated or control site.

**Fig 6:**
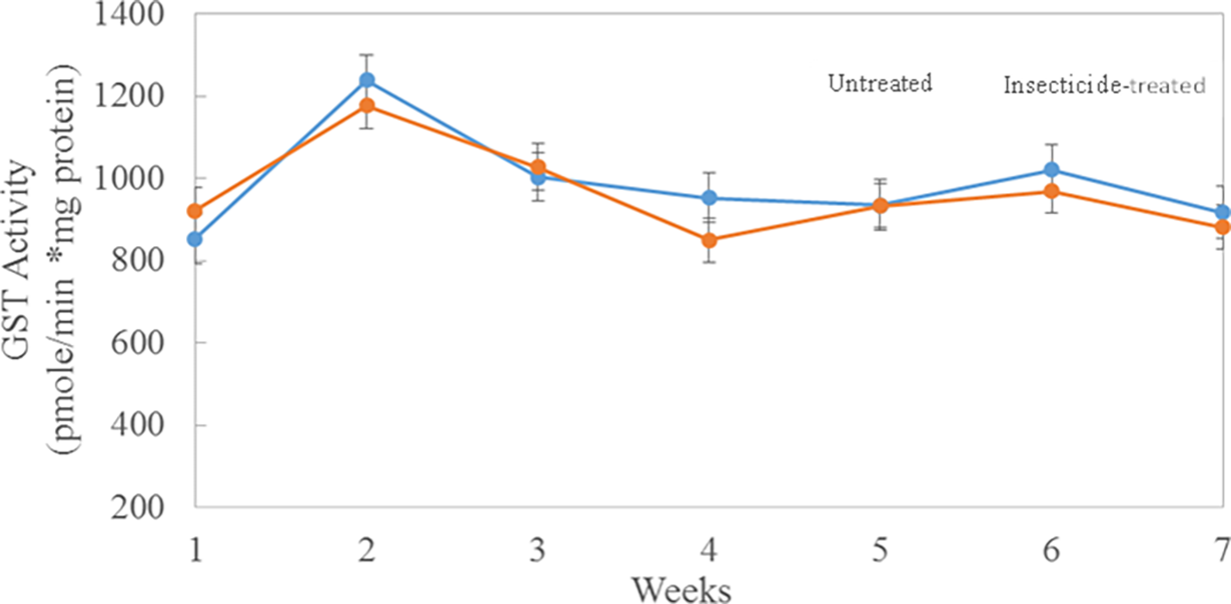
GST activity from bees collected weekly from five insecticide-treated (orange line) or–untreated (blue line) sites. Points represent mean activities (pmole/min*mg protein; ± SEM) from triplicate assays of 10 bees collected from 3 colonies from each treated or control site.

There was no significant effect on enzyme activities in field exposure to insecticides; however, esterase activity was inhibited following topical exposure of bees to a sublethal dose of resmethrin in the laboratory (T_166_ = 4.45 and P = <0.0001; Table 3). In the laboratory, esterase activity decreased by 23%, 12 hr after topical treatment with resmethrin (707.4 μmole/min*mg protein) compared with the control group that was treated with acetone (870.4 μmole/min*mg protein). However, there was no statistically significant difference in GST activities between control and treated groups. In contrast, in the field study, there were no significant effects of insecticide sprays (including resmethrin) on activities of either esterase or GST at control and treated sites (F _1,7_ = 0.08 and P = 0.7902 for esterase activity; F _1,7_ = 0.05 and P = 0.8309 for GST; Table 3). Further, enzyme activities did not differ when measured 2–3 hours before or 10–12 hours after a mosquito control application (Table 3) at the treatment sites (|T| 190 = -0.21 and P = 0.3075 for esterase activity; |T| 190 = 0.7; P = 0.4827 for GST activity). In addition, when sites were analyzed individually, enzyme activities were similar among control and insecticide-treated sites: there were no significant differences in esterase activities among individual sites (Fig 3) except for site 1 (a control site; 1300 μmole/min*mg protein) and site 4 (a treated site; 913.0 μmole/min*mg protein). Similarly, there were no significant differences in GST activities among individual sites (Fig 4) except for site 9 (a control site). For both esterases and GSTs, activities were similar among individual control and treated sites during the seven-week period of the study. Esterase activities peaked at week 2, decreased to week 5, then remained relatively constant through week 7 (Fig 5). Similarly, GST activity also peaked at week 2, declined by week 5, and then remained constant through week 7 (Fig 6). Protein content did not differ (P = 0.213) during the seven-week study for bees in the two treatment groups (4.06 μg/assay for control and 3.96 μg/assay for treated colonies).

**Table 3:** Effect of insecticide exposure on enzyme activities in honey bees from laboratory and field studies.

Experiment	Treatments	n	Enzyme Activity	
			Esterase	GST
Laboratory	Control	3	870 ± 25a	567 ± 24a
	Resmethrin -treated	3	707 ± 26b (-18.74%)	543 ± 26a (-4.23%)
Field	Unsprayed	84	1078 ± 58a	959 ± 48a
	Sprayed	105	1057 ± 51a (-1.95%)	970 ± 43a (+1.15%)
	Pre spray	60	1112 ± 39a	934 ± 20a
	Post spray	60	1093 ± 36a (-1.71%)	931 ± 22a (-0.32%)

Mean activities expressed as μmole/min* mg protein for esterase and pmole/min* mg protein for GST are based on determinations (n) made from individual bees. For the laboratory study, determinations represent mean activities from 10 individual bees assayed on three different days. For the field experiment, determinations were made from groups of 10 individual bees collected weekly from colonies at sprayed or unsprayed sites, or from groups of 10 bees collected 2–3 hours prior (pre-spray) or 10–12 hours after (post spray) an insecticide application. Increase and reduction in enzyme activities compared with control are represented in parenthesis. Positive and negative signs in parenthesis denote increase and decrease in enzyme activity respectively. Significant difference is indicated by different letters within the treatments (P<0.05).

## Discussion

We measured effects of insecticide exposure from urban mosquito abatement efforts on managed honey bee colonies during the most active period of annual spraying. Whereas insecticide exposure of hives was documented, there were no significant effects of these sprays on bee mortality. Throughout the course of our study, we typically saw between 25 to 120 dead bees per colony per week, which is considered natural mortality in bee colonies [39]. A similar study found no effect of aerial spraying of pyrethrin synergized with piperonyl butoxide on mortality of sentinel urban bee colonies in California [6]. Similarly, a semi-field study assessing the impact of truck-based ULV mosquito adulticide applications on honey bees found minimal bee mortality compared to mosquito mortality [40].

In addition to overall weekly mortality, we found few differences in colony health parameters between treated and control sites from the beginning to the end of our study. Whereas we did observe a decrease in the numbers of adult bees in colonies at both treated and control sites, that difference was not statistically significant. It is likely that the small decrease in number of adult bees may be due to the poor resources available for bees during August and September, when the test was conducted. This finding supports previous studies that showed minimal effects of ground applied, ULV sprays of mosquito adulticides in either open or forested areas [11]. Similarly, brood quality increased during the seven week study but did not differ significantly among treated and control sites. This supports results from a previous study [41] in which night time aerial application of the pyrethroids, phenothrin and deltamethrin, had no significant effect on number of adults or brood, or weight of hives in domesticated honey bee colonies. However, in the current study, brood quantity was significantly higher in treated sites (Table 2), but this finding is strongly influenced by two insecticide-exposed colonies (site 2) that had relatively low bee brood populations intially that increased dramatically during the study, so it may not be due to insecticides exposure on the bees.

The inhibition of esterase activity (Table 3) following exposure to a sublethal dose of resmethrin suggests that esterases might be a suitable biomarker of pyrethroid exposure in honey bees. Previous laboratory studies have validated use of esterases as biomarkers for exposure to insecticides (i.e., thiamethoxam and deltametrhin) in honey bee [18,19,42]. Our results support those from a previous laboratory study in which carboxylesterase (CaE-1) activity decreased after treatment with a sublethal dose of deltamethrin on honey bees [43]. Conversely, the lack of inhibition on GST activity following exposure to a maximum sublethal dose of resmethrin in the laboratory suggests that GST may not be a suitable biomarker for pyrethroid exposure, a finding that is in agreement with those from an earlier study [43]. Although resmethrin exposure appears to affect esterase activity, no effect of insecticides on esterase or GST activities was measured in our field study, suggesting that although insecticide residues existed at treated sites (based on recovery of droplets), it is likely that bees were not exposed to them and consequently no significant effect on esterase activities were detected.

In conclusion, minimizing exposure is likely the most important factor that reduces the effects of insecticide applications on honey bees. The mosquito control applications in this study followed label regulations, were conducted between 7:00 pm and 10:00 pm, and utilized properly calibrated equipment [44] Previous researchers have observed higher bee mortality from ground based ULV malathion applications made durng the daytime, whereas night-time applications had no measurable effects on bee mortality [10]. During a night-time application of mosquito adulticides, bees are completely (or mostly) inside the hive, which reduces both exposure to insecticides and acute mortality [45]. However, during hot summer nights, bees may cluster outside the entrance of hives to help increase ventilation. While beekeepers can improve ventilation in hives, this “bearding” behavior may increase the pesticide exposure to those individuals [46]. Additionally, the use of modern spray systems minimizes contamination of the environment, and adoption of high-pressure nozzles has been shown to decrease mortality of bees by more than half [8]. Finally, the laboratory component of the current study validated the use of esterases (but not GST) as a biomarker of pyrethoid exposure in honey bees.

## Supporting information

S1 Appendix Design of dead bee trap: A tool to measure honey bee mortality in colonies. (DOCX)
