# Supplementary material for: Effects of truck-mounted, ultra low volume mosquito adulticides on honey bees (Apis mellifera) in a suburban field setting
Source: PLoS One. 2018 Mar 1;13(3):e0193535. doi: 10.1371/journal.pone.0193535 (PMC5832434; doi:10.1371/journal.pone.0193535)
Supplement: S1 Appendix — (DOCX) [file pone.0193535.s001.docx]

**Supporting Information**

**S1 Appendix. Design of Dead Bee Trap: A Tool to Measure Honey Bee Mortality In Colonies.**

**Design**

We modified a dead bee trap originally designed for measuring honey bee mortality in small, 5-frame “nucleus” colonies (Hendrkisma and Hatrtel [25]). Our modifications allowed the trap to be used on standard, 10-frame Langstroth hives. The trap body is essentially a wooden box that is mounted on the front of the hive and covers the entrance (Fig. S1). Most of the top of the trap is wire screen (hardware cloth) with 6.4-mm (1/4”) openings. Undertaker bees carry dead nest mates out the hive entrance but have difficulty moving the carcasses through the screen. The floor of the trap, 10 cm below the top, has an opening of 27 × 14 cm to accommodate a plastic container (Sterlite #1851; 5.7 liters; 35.6 × 20.3 × 12.4 cm) which collects dead bees that are dropped. The center of the lid of the collection container is removed to leave a 2-cm-wide rim which is glued or screwed to the underside of the trap around the opening in the floor. The collection container is held in place by snapping it into its rim; small holes (4.8 mm; 3/16”) in the bottom of the container allow water to drain. Note that a hive with a trap needs to be elevated to allow space for the collection container. The trap is screwed to the sides of the hive. Any gaps between the trap and the hive (and all other openings to the hive) are sealed with tape to force bees to move through the trap.

We tested the effectiveness of the design by fixing traps onto five hives that housed populous colonies of honey bees. Bees were allowed to acclimatize to the traps for one week, then 100 bees from each hive were collected, killed by freezing, marked on the thorax with yellow enamel paint, and placed under the lid of their own hive. After five days, the numbers of marked bees were counted and used to calculate the efficiency of recovering dead bees in the traps. An average of 95 ± 3% (range 83-100%) of the marked dead bees were recovered from the traps after five days. The high recovery rate compares favorably with results reported for other traps (Table S1).

Table S1. Efficiencies and limitations of different dead bee traps

| Types of Traps | Efficiency | Sample size (n) | Limiatations | References |
| --- | --- | --- | --- | --- |
| Gray Trap | 84% | 15 | Increased artificial mortality, modifies the bee behavior | [1,2] |
| Todd Trap | 90% | Not reported | Difficult to clean debris and is expensive | [3] |
| Munster Dead Bee Trap | 76% | 12 | Low efficiency | [2] |
| Under Basket Trap | 71% | 12 | Low efficiency  Risk of predators | [4,5] |
| Traps for Small Hives | 93± 3% | 9 | Used for only small hives | [6] |
| Modified Hedriksma and Hartel Trap | 95± 3% | 5 | Must be drilled into the bottom frame | This study |

In addition to high efficiency in recovering dead bees, the simple design makes it easy to collect dead bees and clean the trap with minimal interference to the colony. The open top of the trap did not seem to restrict ventilation or cause bees to cluster at the hive entrance. Finally, the trap is very inexpensive to make. These benefits suggest that the trap would be useful in future research that requires monitoring honey bee mortality in the field.

Figure S1. Dead bee trap mounted on a standard, 10-frame Langstroth hive and viewed from the top (a), front (b) and side (c), and obliquely (d). The top, front and bottom of the trap body are constructed of 6.4-mm (1/4”) plywood; the sides are 19-mm (3/4”) thick boards. For reference, the outside dimensions of the trap body are 45.1 cm side to side, 33.0 cm front to back and 10.2 cm top to bottom.


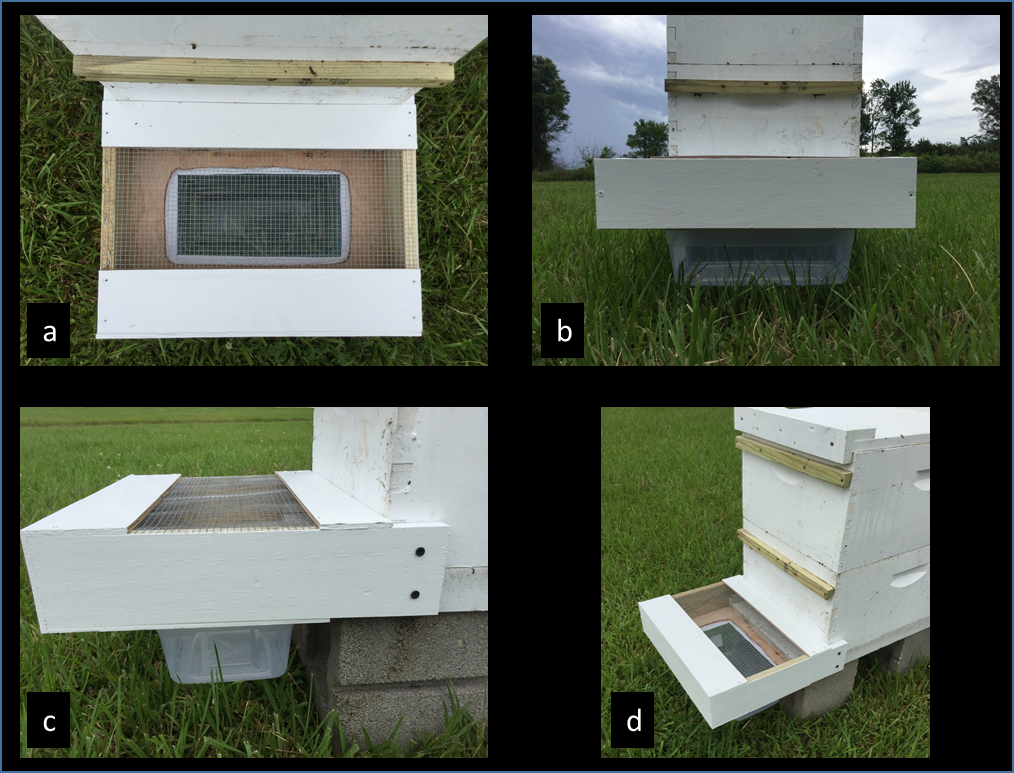


**References**

1. Gary NE. A trap to quantitatively recover dead and abnormal honey bees from the hive. J Econ Entomol. 1960;53(5):782-785.

2. Illies I, Muhlen W, Ducker G, Sachser N. The influence of different bee traps on undertaking behaviour of the honey bee (Apis mellifera) and development of a new trap. Apidol. 2002;33(3):315-326.

3. Atkins E, Todd F, Anderson L. Honey bee field research aided by Todd dead bee hive entrance trap. Calif Agric. 1970;24(10): 12-13.

4. Accorti M, Luti F, Tarducci F. Methods for collecting data on natural mortality in bee. Ethol Ecol Evol. 1991;3(sup 1):123-126.

5. Porrini C, Sabatini AG, Girotti S, Fini F, Monaco L, Celli G, et al. The death of honey bees and environmental pollution by pesticides: the honey bees as biological indicators. Bull Insectol. 2003;56(1):147-152.

6. Hendriksma HP, Härtel S. A simple trap to measure worker bee mortality in small test colonies. J Apicult Res. 2010;49: 215-217.
